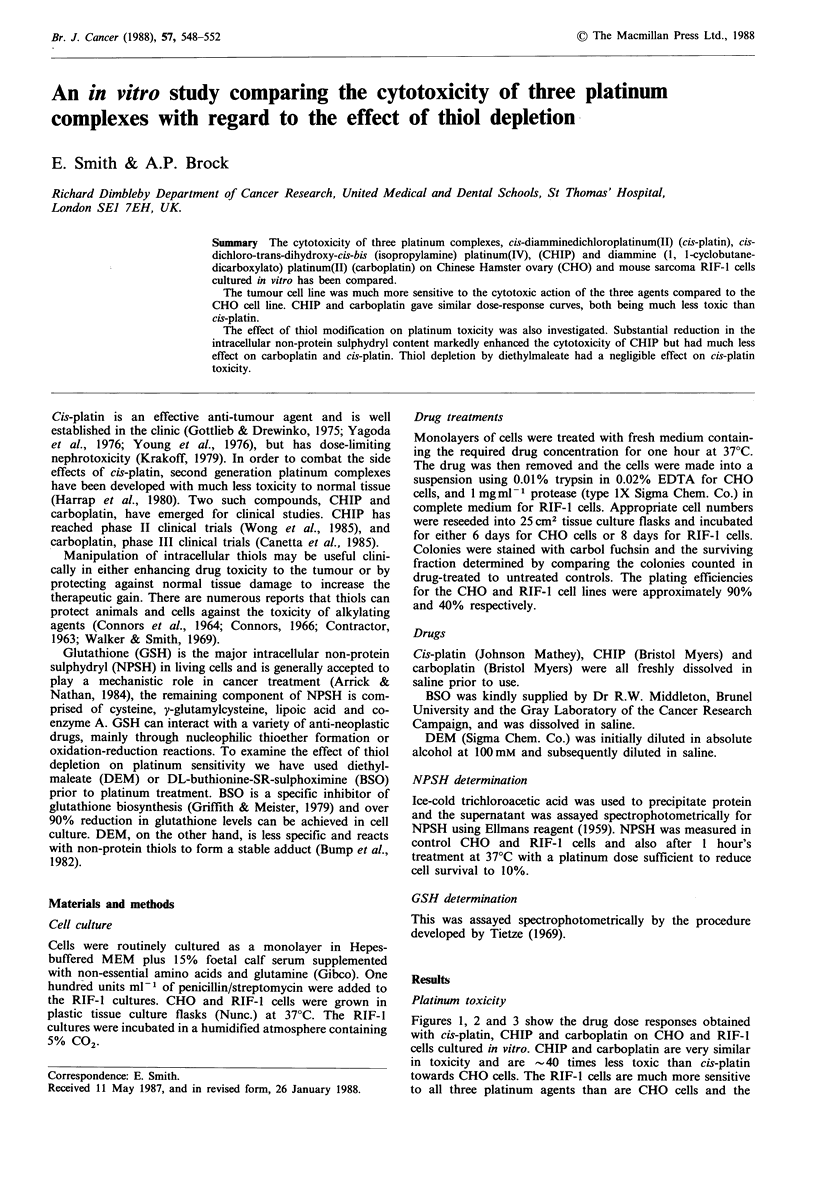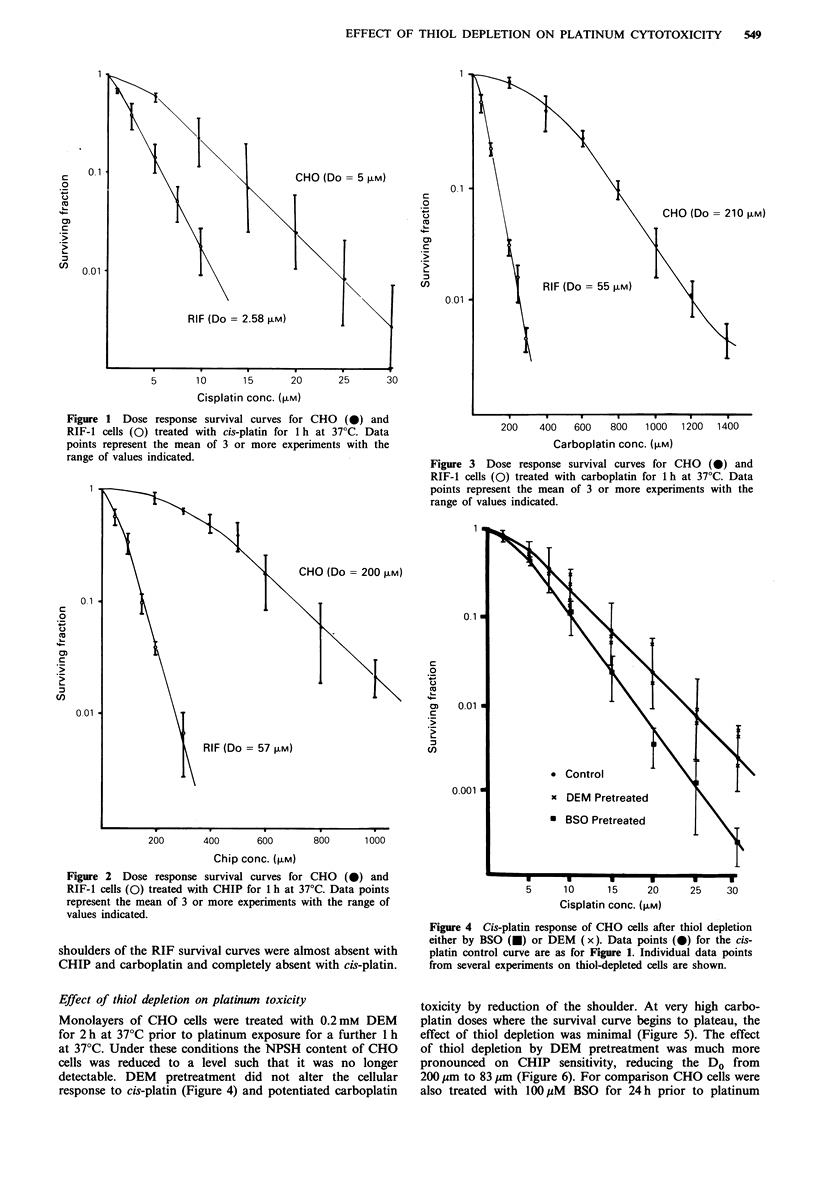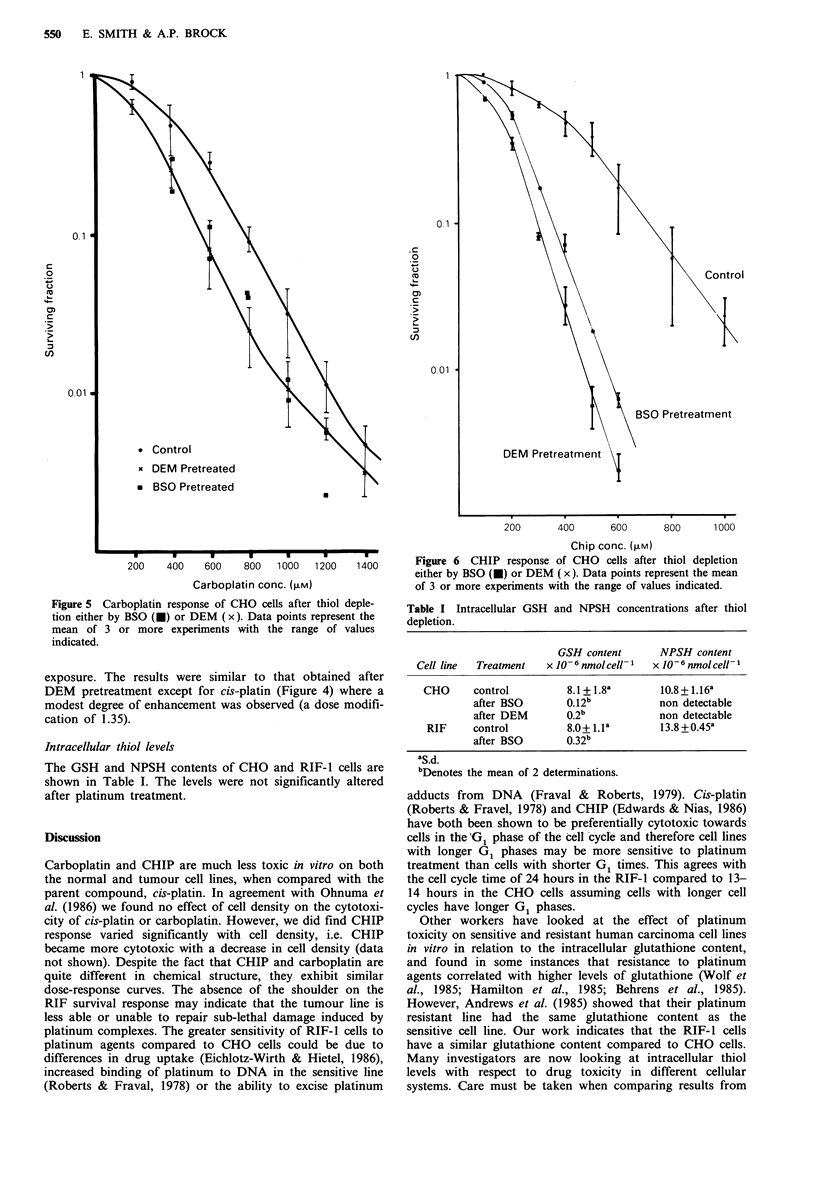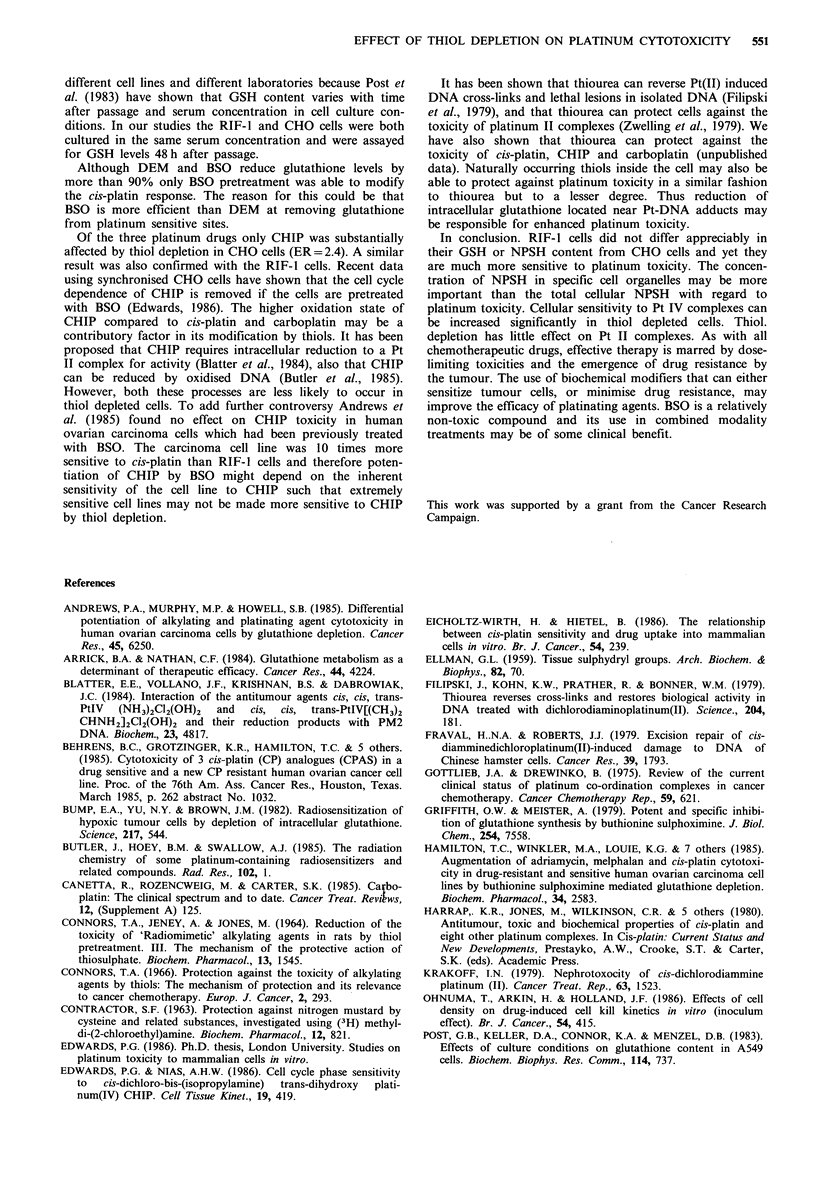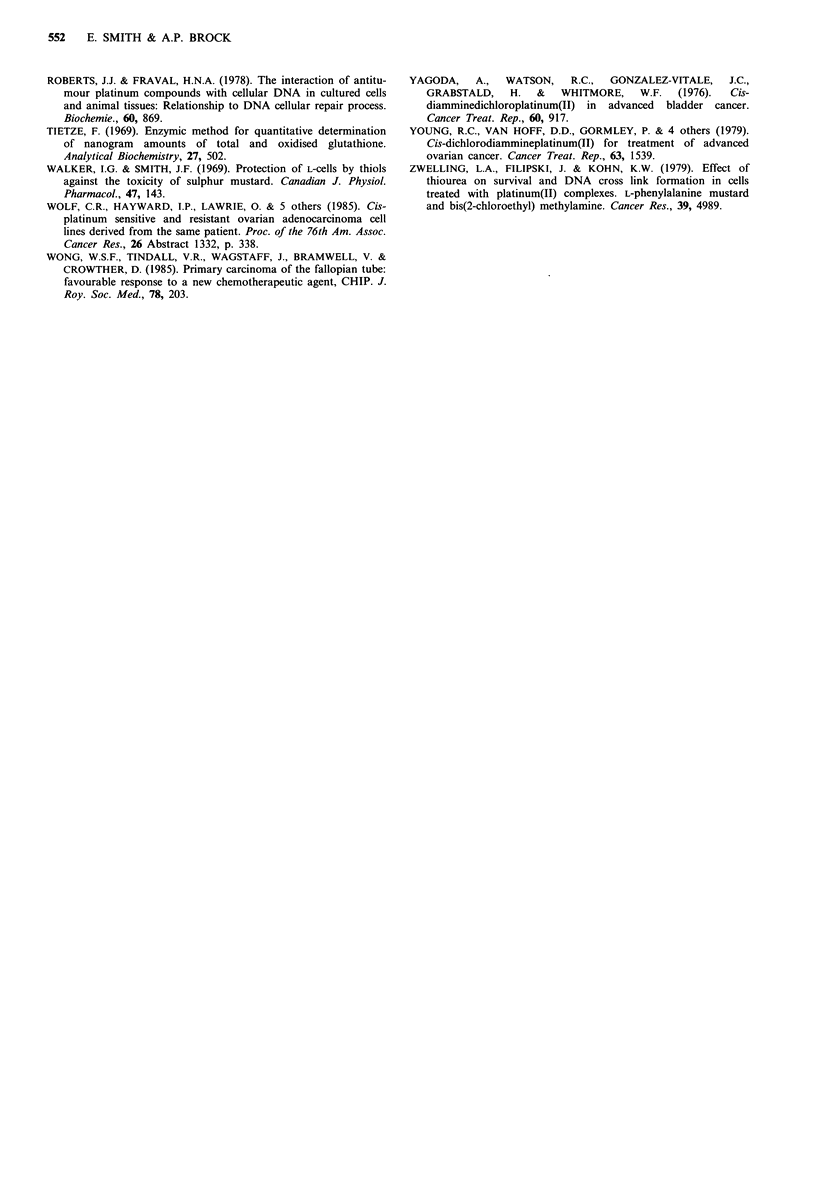# An in vitro study comparing the cytotoxicity of three platinum complexes with regard to the effect of thiol depletion.

**DOI:** 10.1038/bjc.1988.126

**Published:** 1988-06

**Authors:** E. Smith, A. P. Brock

**Affiliations:** Richard Dimbleby Department of Cancer Research, United Medical School, St Thomas' Hospital, London, UK.

## Abstract

The cytotoxicity of three platinum complexes, cis-diamminedichloroplatinum(II) (cis-platin), cis-dichloro-trans-dihydroxy-cis-bis (isopropylamine) platinum(IV), (CHIP) and diammine (1, 1-cyclobutane-dicarboxylato) platinum(II) (carboplatin) on Chinese Hamster ovary (CHO) and mouse sarcoma RIF-1 cells cultured in vitro has been compared. The tumour cell line was much more sensitive to the cytotoxic action of the three agents compared to the CHO cell line. CHIP and carboplatin gave similar dose-response curves, both being much less toxic than cis-platin. The effect of thiol modification on platinum toxicity was also investigated. Substantial reduction in the intracellular non-protein sulphydryl content markedly enhanced the cytotoxicity of CHIP but had much less effect on carboplatin and cis-platin. Thiol depletion by diethylmaleate had a negligible effect on cis-platin toxicity.


Br. J. Cancer (1988), 57, 548–552 © The Macmillan Press Ltd., 1988

### An *in vitro* study comparing the cytotoxicity of three platinum complexes with regard to the effect of thiol depletion

E. Smith & A.P. Brock

*Richard Dimbleby Department of Cancer Research, United Medical and Dental Schools, St Thomas' Hospital, London SE1 7EH, UK.*

**Summary** The cytotoxicity of three platinum complexes, *cis*-diamminedichloroplatinum(II) (*cis*-platin), *cis*-dichloro-trans-dihydroxy-*cis-bis* (isopropylamine) platinum(IV), (CHIP) and diammine (1, 1-cyclobutane-dicarboxylato) platinum(II) (carboplatin) on Chinese Hamster ovary (CHO) and mouse sarcoma RIF-1 cells cultured *in vitro* has been compared.

The tumour cell line was much more sensitive to the cytotoxic action of the three agents compared to the CHO cell line. CHIP and carboplatin gave similar dose-response curves, both being much less toxic than *cis*-platin.

The effect of thiol modification on platinum toxicity was also investigated. Substantial reduction in the intracellular non-protein sulphydryl content markedly enhanced the cytotoxicity of CHIP but had much less effect on carboplatin and *cis*-platin. Thiol depletion by diethylmaleate had a negligible effect on *cis*-platin toxicity.

*Cis*-platin is an effective anti-tumour agent and is well established in the clinic (Gottlieb & Drewinko, 1975; Yagoda *et al.*, 1976; Young *et al.*, 1976), but has dose-limiting nephrotoxicity (Krakoff, 1979). In order to combat the side effects of *cis*-platin, second generation platinum complexes have been developed with much less toxicity to normal tissue (Harrap *et al.*, 1980). Two such compounds, CHIP and carboplatin, have emerged for clinical studies. CHIP has reached phase II clinical trials (Wong *et al.*, 1985), and carboplatin, phase III clinical trials (Canetta *et al.*, 1985).

Manipulation of intracellular thiols may be useful clinically in either enhancing drug toxicity to the tumour or by protecting against normal tissue damage to increase the therapeutic gain. There are numerous reports that thiols can protect animals and cells against the toxicity of alkylating agents (Connors *et al.*, 1964; Connors, 1966; Contractor, 1963; Walker & Smith, 1969).

Glutathione (GSH) is the major intracellular non-protein sulphydryl (NPSH) in living cells and is generally accepted to play a mechanistic role in cancer treatment (Arrick & Nathan, 1984), the remaining component of NPSH is comprised of cysteine, $\gamma$-glutamylcysteine, lipoic acid and co-enzyme A. GSH can interact with a variety of anti-neoplastic drugs, mainly through nucleophilic thioether formation or oxidation-reduction reactions. To examine the effect of thiol depletion on platinum sensitivity we have used diethylmaleate (DEM) or DL-buthionine-SR-sulphoximine (BSO) prior to platinum treatment. BSO is a specific inhibitor of glutathione biosynthesis (Griffith & Meister, 1979) and over 90% reduction in glutathione levels can be achieved in cell culture. DEM, on the other hand, is less specific and reacts with non-protein thiols to form a stable adduct (Bump *et al.*, 1982).

### Materials and methods

### Cell culture

Cells were routinely cultured as a monolayer in Hepes-buffered MEM plus 15% foetal calf serum supplemented with non-essential amino acids and glutamine (Gibco). One hundred units $ml^{-1}$ of penicillin/streptomycin were added to the RIF-1 cultures. CHO and RIF-1 cells were grown in plastic tissue culture flasks (Nunc.) at 37°C. The RIF-1 cultures were incubated in a humidified atmosphere containing 5% $CO_2$.

Correspondence: E. Smith.
Received 11 May 1987, and in revised form, 26 January 1988.

### Drug treatments

Monolayers of cells were treated with fresh medium containing the required drug concentration for one hour at 37°C. The drug was then removed and the cells were made into a suspension using 0.01% trypsin in 0.02% EDTA for CHO cells, and 1 mg $ml^{-1}$ protease (type 1X Sigma Chem. Co.) in complete medium for RIF-1 cells. Appropriate cell numbers were reseeded into 25 $cm^2$ tissue culture flasks and incubated for either 6 days for CHO cells or 8 days for RIF-1 cells. Colonies were stained with carbol fuchsin and the surviving fraction determined by comparing the colonies counted in drug-treated to untreated controls. The plating efficiencies for the CHO and RIF-1 cell lines were approximately 90% and 40% respectively.

### Drugs

*Cis*-platin (Johnson Mathey), CHIP (Bristol Myers) and carboplatin (Bristol Myers) were all freshly dissolved in saline prior to use.

BSO was kindly supplied by Dr R.W. Middleton, Brunel University and the Gray Laboratory of the Cancer Research Campaign, and was dissolved in saline.

DEM (Sigma Chem. Co.) was initially diluted in absolute alcohol at 100 mM and subsequently diluted in saline.

### NPSH determination

Ice-cold trichloroacetic acid was used to precipitate protein and the supernatant was assayed spectrophotometrically for NPSH using Ellmans reagent (1959). NPSH was measured in control CHO and RIF-1 cells and also after 1 hour's treatment at 37°C with a platinum dose sufficient to reduce cell survival to 10%.

### GSH determination

This was assayed spectrophotometrically by the procedure developed by Tietze (1969).

### Results

### Platinum toxicity

Figures 1, 2 and 3 show the drug dose responses obtained with *cis*-platin, CHIP and carboplatin on CHO and RIF-1 cells cultured *in vitro*. CHIP and carboplatin are very similar in toxicity and are ~40 times less toxic than *cis*-platin towards CHO cells. The RIF-1 cells are much more sensitive to all three platinum agents than are CHO cells and the

**Figure 1** Dose response survival curves for CHO (●) and RIF-1 cells (○) treated with *cis*-platin for 1 h at 37°C. Data points represent the mean of 3 or more experiments with the range of values indicated.

**Figure 2** Dose response survival curves for CHO (●) and RIF-1 cells (○) treated with CHIP for 1 h at 37°C. Data points represent the mean of 3 or more experiments with the range of values indicated.

**Figure 3** Dose response survival curves for CHO (●) and RIF-1 cells (○) treated with carboplatin for 1 h at 37°C. Data points represent the mean of 3 or more experiments with the range of values indicated.

**Figure 4** *Cis*-platin response of CHO cells after thiol depletion either by BSO (■) or DEM (×). Data points (●) for the *cis*-platin control curve are as for **Figure 1**. Individual data points from several experiments on thiol-depleted cells are shown.

shoulders of the RIF survival curves were almost absent with CHIP and carboplatin and completely absent with *cis*-platin.

*Effect of thiol depletion on platinum toxicity*

Monolayers of CHO cells were treated with 0.2 mM DEM for 2 h at 37°C prior to platinum exposure for a further 1 h at 37°C. Under these conditions the NPSH content of CHO cells was reduced to a level such that it was no longer detectable. DEM pretreatment did not alter the cellular response to *cis*-platin (Figure 4) and potentiated carboplatin toxicity by reduction of the shoulder. At very high carboplatin doses where the survival curve begins to plateau, the effect of thiol depletion was minimal (Figure 5). The effect of thiol depletion by DEM pretreatment was much more pronounced on CHIP sensitivity, reducing the $D_0$ from 200 μm to 83 μm (Figure 6). For comparison CHO cells were also treated with 100 μM BSO for 24 h prior to platinum

**Figure 5** Carboplatin response of CHO cells after thiol depletion either by BSO (■) or DEM (×). Data points represent the mean of 3 or more experiments with the range of values indicated.

**Figure 6** CHIP response of CHO cells after thiol depletion either by BSO (■) or DEM (×). Data points represent the mean of 3 or more experiments with the range of values indicated.

**Table I** Intracellular GSH and NPSH concentrations after thiol depletion.

| *Cell line* | *Treatment* | *GSH content* $\times 10^{-6}$ *nmol cell*$^{-1}$ | *NPSH content* $\times 10^{-6}$ *nmol cell*$^{-1}$ |
|---|---|---|---|
| CHO | control | $8.1 \pm 1.8^a$ | $10.8 \pm 1.16^a$ |
| | after BSO | $0.12^b$ | non detectable |
| | after DEM | $0.2^b$ | non detectable |
| RIF | control | $8.0 \pm 1.1^a$ | $13.8 \pm 0.45^a$ |
| | after BSO | $0.32^b$ | |

[a]S.d.
[b]Denotes the mean of 2 determinations.

exposure. The results were similar to that obtained after DEM pretreatment except for *cis*-platin (Figure 4) where a modest degree of enhancement was observed (a dose modification of 1.35).

*Intracellular thiol levels*

The GSH and NPSH contents of CHO and RIF-1 cells are shown in Table I. The levels were not significantly altered after platinum treatment.

### Discussion

Carboplatin and CHIP are much less toxic *in vitro* on both the normal and tumour cell lines, when compared with the parent compound, *cis*-platin. In agreement with Ohnuma *et al.* (1986) we found no effect of cell density on the cytotoxicity of *cis*-platin or carboplatin. However, we did find CHIP response varied significantly with cell density, i.e. CHIP became more cytotoxic with a decrease in cell density (data not shown). Despite the fact that CHIP and carboplatin are quite different in chemical structure, they exhibit similar dose-response curves. The absence of the shoulder on the RIF survival response may indicate that the tumour line is less able or unable to repair sub-lethal damage induced by platinum complexes. The greater sensitivity of RIF-1 cells to platinum agents compared to CHO cells could be due to differences in drug uptake (Eichlotz-Wirth & Hietel, 1986), increased binding of platinum to DNA in the sensitive line (Roberts & Fraval, 1978) or the ability to excise platinum adducts from DNA (Fraval & Roberts, 1979). *Cis*-platin (Roberts & Fravel, 1978) and CHIP (Edwards & Nias, 1986) have both been shown to be preferentially cytotoxic towards cells in the $G_1$ phase of the cell cycle and therefore cell lines with longer $G_1$ phases may be more sensitive to platinum treatment than cells with shorter $G_1$ times. This agrees with the cell cycle time of 24 hours in the RIF-1 compared to 13–14 hours in the CHO cells assuming cells with longer cell cycles have longer $G_1$ phases.

Other workers have looked at the effect of platinum toxicity on sensitive and resistant human carcinoma cell lines *in vitro* in relation to the intracellular glutathione content, and found in some instances that resistance to platinum agents correlated with higher levels of glutathione (Wolf *et al.*, 1985; Hamilton *et al.*, 1985; Behrens *et al.*, 1985). However, Andrews *et al.* (1985) showed that their platinum resistant line had the same glutathione content as the sensitive cell line. Our work indicates that the RIF-1 cells have a similar glutathione content compared to CHO cells. Many investigators are now looking at intracellular thiol levels with respect to drug toxicity in different cellular systems. Care must be taken when comparing results from different cell lines and different laboratories because Post *et al.* (1983) have shown that GSH content varies with time after passage and serum concentration in cell culture conditions. In our studies the RIF-1 and CHO cells were both cultured in the same serum concentration and were assayed for GSH levels 48 h after passage.

Although DEM and BSO reduce glutathione levels by more than 90% only BSO pretreatment was able to modify the *cis*-platin response. The reason for this could be that BSO is more efficient than DEM at removing glutathione from platinum sensitive sites.

Of the three platinum drugs only CHIP was substantially affected by thiol depletion in CHO cells (ER = 2.4). A similar result was also confirmed with the RIF-1 cells. Recent data using synchronised CHO cells have shown that the cell cycle dependence of CHIP is removed if the cells are pretreated with BSO (Edwards, 1986). The higher oxidation state of CHIP compared to *cis*-platin and carboplatin may be a contributory factor in its modification by thiols. It has been proposed that CHIP requires intracellular reduction to a Pt II complex for activity (Blatter *et al.*, 1984), also that CHIP can be reduced by oxidised DNA (Butler *et al.*, 1985). However, both these processes are less likely to occur in thiol depleted cells. To add further controversy Andrews *et al.* (1985) found no effect on CHIP toxicity in human ovarian carcinoma cells which had been previously treated with BSO. The carcinoma cell line was 10 times more sensitive to *cis*-platin than RIF-1 cells and therefore potentiation of CHIP by BSO might depend on the inherent sensitivity of the cell line to CHIP such that extremely sensitive cell lines may not be made more sensitive to CHIP by thiol depletion.

It has been shown that thiourea can reverse Pt(II) induced DNA cross-links and lethal lesions in isolated DNA (Filipski *et al.*, 1979), and that thiourea can protect cells against the toxicity of platinum II complexes (Zwelling *et al.*, 1979). We have also shown that thiourea can protect against the toxicity of *cis*-platin, CHIP and carboplatin (unpublished data). Naturally occurring thiols inside the cell may also be able to protect against platinum toxicity in a similar fashion to thiourea but to a lesser degree. Thus reduction of intracellular glutathione located near Pt-DNA adducts may be responsible for enhanced platinum toxicity.

In conclusion. RIF-1 cells did not differ appreciably in their GSH or NPSH content from CHO cells and yet they are much more sensitive to platinum toxicity. The concentration of NPSH in specific cell organelles may be more important than the total cellular NPSH with regard to platinum toxicity. Cellular sensitivity to Pt IV complexes can be increased significantly in thiol depleted cells. Thiol. depletion has little effect on Pt II complexes. As with all chemotherapeutic drugs, effective therapy is marred by dose-limiting toxicities and the emergence of drug resistance by the tumour. The use of biochemical modifiers that can either sensitize tumour cells, or minimise drug resistance, may improve the efficacy of platinating agents. BSO is a relatively non-toxic compound and its use in combined modality treatments may be of some clinical benefit.

This work was supported by a grant from the Cancer Research Campaign.

### References

ANDREWS, P.A., MURPHY, M.P. & HOWELL, S.B. (1985). Differential potentiation of alkylating and platinating agent cytotoxicity in human ovarian carcinoma cells by glutathione depletion. *Cancer Res.*, **45**, 6250.

ARRICK, B.A. & NATHAN, C.F. (1984). Glutathione metabolism as a determinant of therapeutic efficacy. *Cancer Res.*, **44**, 4224.

BLATTER, E.E., VOLLANO, J.F., KRISHNAN, B.S. & DABROWIAK, J.C. (1984). Interaction of the antitumour agents *cis*, *cis*, trans-PtIV $(NH_3)_2Cl_2(OH)_2$ and *cis*, *cis*, trans-PtIV$[(CH_3)_2CHNH_2]_2Cl_2(OH)_2$ and their reduction products with PM2 DNA. *Biochem.*, **23**, 4817.

BEHRENS, B.C., GROTZINGER, K.R., HAMILTON, T.C. & 5 others. (1985). Cytotoxicity of 3 *cis*-platin (CP) analogues (CPAS) in a drug sensitive and a new CP resistant human ovarian cancer cell line. Proc. of the 76th Am. Ass. Cancer Res., Houston, Texas. March 1985, p. 262 abstract No. 1032.

BUMP, E.A., YU, N.Y. & BROWN, J.M. (1982). Radiosensitization of hypoxic tumour cells by depletion of intracellular glutathione. *Science*, **217**, 544.

BUTLER, J., HOEY, B.M. & SWALLOW, A.J. (1985). The radiation chemistry of some platinum-containing radiosensitizers and related compounds. *Rad. Res.*, **102**, 1.

CANETTA, R., ROZENCWEIG, M. & CARTER, S.K. (1985). Carboplatin: The clinical spectrum and to date. *Cancer Treat. Reviews*, **12**, (Supplement A) 125.

CONNORS, T.A., JENEY, A. & JONES, M. (1964). Reduction of the toxicity of 'Radiomimetic' alkylating agents in rats by thiol pretreatment. III. The mechanism of the protective action of thiosulphate. *Biochem. Pharmacol.*, **13**, 1545.

CONNORS, T.A. (1966). Protection against the toxicity of alkylating agents by thiols: The mechanism of protection and its relevance to cancer chemotherapy. *Europ. J. Cancer*, **2**, 293.

CONTRACTOR, S.F. (1963). Protection against nitrogen mustard by cysteine and related substances, investigated using ($^3$H) methyl-di-(2-chloroethyl)amine. *Biochem. Pharmacol.*, **12**, 821.

EDWARDS, P.G. (1986). Ph.D. thesis, London University. Studies on platinum toxicity to mammalian cells *in vitro*.

EDWARDS, P.G. & NIAS, A.H.W. (1986). Cell cycle phase sensitivity to *cis*-dichloro-bis-(isopropylamine) trans-dihydroxy platinum(IV) CHIP. *Cell Tissue Kinet.*, **19**, 419.

EICHOLTZ-WIRTH, H. & HIETEL, B. (1986). The relationship between *cis*-platin sensitivity and drug uptake into mammalian cells *in vitro*. *Br. J. Cancer.*, **54**, 239.

ELLMAN, G.L. (1959). Tissue sulphydryl groups. *Arch. Biochem. & Biophys.*, **82**, 70.

FILIPSKI, J., KOHN, K.W., PRATHER, R. & BONNER, W.M. (1979). Thiourea reverses cross-links and restores biological activity in DNA treated with dichlorodiaminoplatinum(II). *Science.*, **204**, 181.

FRAVAL, H..N.A. & ROBERTS, J.J. (1979. Excision repair of *cis*-diamminedichloroplatinum(II)-induced damage to DNA of Chinese hamster cells. *Cancer Res.*, **39**, 1793.

GOTTLIEB, J.A. & DREWINKO, B. (1975). Review of the current clinical status of platinum co-ordination complexes in cancer chemotherapy. *Cancer Chemotherapy Rep.*, **59**, 621.

GRIFFITH, O.W. & MEISTER, A. (1979). Potent and specific inhibition of glutathione synthesis by buthionine sulphoximine. *J. Biol. Chem.*, **254**, 7558.

HAMILTON, T.C., WINKLER, M.A., LOUIE, K.G. & 7 others (1985). Augmentation of adriamycin, melphalan and *cis*-platin cytotoxicity in drug-resistant and sensitive human ovarian carcinoma cell lines by buthionine sulphoximine mediated glutathione depletion. *Biochem. Pharmacol.*, **34**, 2583.

HARRAP,. K.R., JONES, M., WILKINSON, C.R. & 5 others (1980). Antitumour, toxic and biochemical properties of *cis*-platin and eight other platinum complexes. In Cis-*platin: Current Status and New Developments*, Prestayko, A.W., Crooke, S.T. & Carter, S.K. (eds). Academic Press.

KRAKOFF, I.N. (1979). Nephrotoxocity of *cis*-dichlorodiammine platinum (II). *Cancer Treat. Rep.*, **63**, 1523.

OHNUMA, T., ARKIN, H. & HOLLAND, J.F. (1986). Effects of cell density on drug-induced cell kill kinetics *in vitro* (inoculum effect). *Br. J. Cancer.*, **54**, 415.

POST, G.B., KELLER, D.A., CONNOR, K.A. & MENZEL, D.B. (1983). Effects of culture conditions on glutathione content in A549 cells. *Biochem. Biophys. Res. Comm.*, **114**, 737.

ROBERTS, J.J. & FRAVAL, H.N.A. (1978). The interaction of antitumour platinum compounds with cellular DNA in cultured cells and animal tissues: Relationship to DNA cellular repair process. *Biochemie.*, **60**, 869.

TIETZE, F. (1969). Enzymic method for quantitative determination of nanogram amounts of total and oxidised glutathione. *Analytical Biochemistry*, **27**, 502.

WALKER, I.G. & SMITH, J.F. (1969). Protection of L-cells by thiols against the toxicity of sulphur mustard. *Canadian J. Physiol. Pharmacol.*, **47**, 143.

WOLF, C.R., HAYWARD, I.P., LAWRIE, O. & 5 others (1985). *Cis*-platinum sensitive and resistant ovarian adenocarcinoma cell lines derived from the same patient. *Proc. of the 76th Am. Assoc. Cancer Res.*, **26** Abstract 1332, p. 338.

WONG, W.S.F., TINDALL, V.R., WAGSTAFF, J., BRAMWELL, V. & CROWTHER, D. (1985). Primary carcinoma of the fallopian tube: favourable response to a new chemotherapeutic agent, CHIP. *J. Roy. Soc. Med.*, **78**, 203.

YAGODA, A., WATSON, R.C., GONZALEZ-VITALE, J.C., GRABSTALD, H. & WHITMORE, W.F. (1976). *Cis*-diamminedichloroplatinum(II) in advanced bladder cancer. *Cancer Treat. Rep.*, **60**, 917.

YOUNG, R.C., VAN HOFF, D.D., GORMLEY, P. & 4 others (1979). *Cis*-dichlorodiammineplatinum(II) for treatment of advanced ovarian cancer. *Cancer Treat. Rep.*, **63**, 1539.

ZWELLING, L.A., FILIPSKI, J. & KOHN, K.W. (1979). Effect of thiourea on survival and DNA cross link formation in cells treated with platinum(II) complexes. L-phenylalanine mustard and bis(2-chloroethyl) methylamine. *Cancer Res.*, **39**, 4989.